# Looking for protein stabilizing drugs with thermal shift assay

**DOI:** 10.1002/dta.1798

**Published:** 2015-04-05

**Authors:** Giuseppina Andreotti, Maria Monticelli, Maria Vittoria Cubellis

**Affiliations:** ^1^ Istituto di Chimica Biomolecolare –CNR Pozzuoli Italy; ^2^ Dipartimento di Biologia Università Federico II Napoli Italy

**Keywords:** rare disease, pharmacological chaperones, thermal shift assay

## Abstract

Thermal shift assay can be used for the high‐throughput screening of pharmacological chaperones. These drugs are small molecules that bind a mutant protein and stabilize it. We demonstrated the robustness, reproducibility and versatility of the method using two molecules that are in clinical trial for Fabry or Pompe disease, Deoxygalactonojirimycin and N‐Butyldeoxynojirimycin, and their target enzymes, lysosomal alpha‐galactosidaseA and alpha‐glucosidase, as test cases. We assessed the influence of solvents and of scanning rate on the measures. We showed that a value that is equivalent to the melting temperature can be obtained by the first derivatives of raw data. We discuss the advantages of the method and the precaution to be taken in running the experiments. © 2015 The Authors *Drug Testing and Analysis* Published by John Wiley & Sons Ltd.

A good share of the mutations associated with human diseases causes protein destabilization.^1^ Pharmacological chaperones (PCs) stabilize proteins that retain the essential residues needed for activity, but have become unstable upon mutation and are degraded by the quality control system of the cell. Some PCs are already in clinical trial.^2^

Mutations in the gene encoding lysosomal alpha‐galactosidaseA and lysosomal alpha‐glucosidase are associated with Fabry and Pompe diseases, respectively. Deoxygalactonojirimycin (DGJ) at low concentration effectively increases mutant lysosomal alpha‐galactosidaseA activity (for a review please consult Fabry_CEP^3^ and references therein) while N‐Butyldeoxynojirimycin (NB‐DNJ) increases mutant lysosomal alpha‐glucosidase activity.^4^ PCs are often described as reversible inhibitors used at sub‐inhibitory concentration. This description is deceptive, because the ability to reduce the activity of the target enzyme is neither required nor desirable for the therapeutic action. The first effective drugs, as DGJ or NB‐DNJ, were indeed found among inhibitors, but this was due to practical reasons, specifically the lack of a systematic screening. The best definition for these medicines should be ‘thermodynamic drugs’ because altogether they are ligands that preferentially bind the folded state of a protein. For this reason a screening method based on protein stabilization is more appropriate to find PC than a method based on enzyme inhibition. Thermal shift assay (TSA), which has been described by several authors,^5^, ^6^, ^7^, ^8^, ^9^, ^10^ has found different applications not only for the identification of enzyme inhibitors, but also for activators and allosteric ligands.^11^, ^12^

We carried out TSA with the StepOne™ Real‐Time PCR Systems. The protein (0.1–0.5 mg/mL final concentration) was equilibrated in the appropriate buffer (Na‐Hepes 20 mM, NaCl 150 mM, pH 7.4 or Na‐acetate 20 mM, NaCl 150 mM, pH 4.2 or Na‐acetate 20 mM, NaCl 150 mM pH 5.2) with Sypro Orange 2.5X (Invitrogen Molecular Probes, lifetechnologies.com), both with and without ligands. The samples were distributed in PCR multi‐strip (0.025 mL in each well), sealed with cap strips and heated from 25 to 85° at 0.5°C/min or 1°C/min, with increments of 0.3 or 0.6°C, respectively.

TSA requires purified proteins for the screening. In a few cases it was possible to over‐express and purify the mutant proteins,^13^ but in general this is unpractical. Apparently this represents a limitation of the method. However if a ligand is able to stabilize mutant forms, it can also stabilize the wild type protein, which can conveniently replace mutants for screening purposes. Human wild type recombinant lysosomal alpha‐galactosidaseA and alpha‐glucosidase are commercially available with the names of Fabrazyme® and Myozyme® (Genzyme Corporation, Cambridge, MA, USA), respectively. In Figure 1, panels A, B, and C, we show the melting profiles of Fabrazyme® recorded at 0.5°C/min with or without ligands, (0.04 mM DGJ (SIGMA, Milan, Italy) or 100 mM galactose). Raw data are unpractical for comparison. The unfolded fraction can be calculated as fu(T) = f(T)‐fn(T)/fd(T)‐fn(T) where f(T) is the fluorescence at temperature T, fn(T), and fd(T) are the values of fluorescence extrapolated at temperature T from the native and unfolded regions of the melting profile (data not shown) or, more simplistically, normalizing the melting profiles using the equation fu(T) = f(T)‐fn/fd‐fn, where fn represents the minimum value of the fluorescence before the transition and fd represents the maximum value after the transition (Figure 1, panels A,B,C,F; and Figure 2, panels A,B,C,F). From these curves we calculated the midpoint of the protein melting transition, T_0.5_ and observed that the effect of the simplification on T_0.5_ is negligible if compared with variability, on average ±1°C, among our replicas. In order to assess the reproducibility of the measures and the influence of the technique employed, we compared our results (T_0.5_=48°C neutral pH no ligand; T_0.5_=56°C acidic lysosomal pH no ligand; T_0.5_=61°C neutral pH plus DGJ; T_0.5_=70°C acidic lysosomal pH plus DGJ) with those obtained by Valenzano *et al*.^14^ (T_0.5_=47°C neutral pH no ligand; T_0.5_=58°C acidic lysosomal pH no ligand; T_0.5_=58°C neutral pH plus DGJ; T_0.5_=72°C acidic lysosomal pH plus DGJ) exploiting TSA or by Petsko *et al*.^15^ (T_0.5_=48.0°C neutral pH no ligand; T_0.5_=60.2°C acidic lysosomal pH no ligand; T_0.5_=60.6°C neutral pH plus DGJ; T_0.5_=73.5°C acidic lysosomal pH plus DGJ) exploiting differential scanning calorimetry. Taken together the three sets of experiments cover a broad range of protein concentrations (10 μM our data, 2 μM Valenzano *et al*.^14^ 47.5 μM or 8.6 μM Petsko *et al*.^15^) and scanning rates (0.5°C/min our data, 1.0°C/min Valenzano *et al*.^14^ 1.0°C/min or 1.5°C/min Petsko *et al*.^15^). Denaturation induced during TSA is not a reversible process and the parameters which are measured cannot be defined as thermodynamic properties of the system, nonetheless the coincidence of T_0.5_ measured by three groups with different experimental procedures allows us to consider TSA as a sufficiently robust method to assay lysosomal alpha‐galactosidaseA stability under different conditions. The parallelism of the results obtainable with TSA and with calorimetry can be pushed further. Examining the melting curve profiles, we observed that the fraction of unfolded apo‐enzyme increases from 10% to 90% over 5–6 °C both at pH 7.4 (Figure 1, panel C) and 5.2 (Figure 1, panel B) in a highly cooperative fashion whereas a more complex unfolding process occurs in the presence of DGJ. This confirms the results obtained by Petsko *et al*.^15^ who found a coincidence of calorimetric and van't Hoff curves for the free enzyme, but not for the complexed enzyme and suggested that ligand binding leads to the preferential stabilization of the TIM barrel domain where binding takes place. The effect is more evident at pH7.4 than at pH 5.2 both with scanning calorimetry^15^ and with TSA. TSA is useful to compare the stabilizing effect of different ligands and infer the binding modes. Galactose has a lesser stabilizing effect than DGJ; this reflects the fact that galactose has lower affinity for alpha‐galactosidaseA than DGJ, K_i_ 16 mM and 39 nM, respectively.^16^ Melting temperature variations, ΔT_0.5_=T_0.5DGJ_‐ T_0.5galactose_, are not dependent on pH over the range explored (Figure 1, panel D). DGJ and galactose have a different heteroatom in the ring, N or O, respectively, with a different protonation state. If a titrable residue was responsible for the higher affinity of DGJ,^16^ it would be expected that ΔT_0.5_ depended on pH. Experiments at different pH are important to show that both DGJ and galactose are not the optimal chaperones for Fabry disease, because they bind the enzyme under neutral and acidic conditions. These molecules in fact are reversible inhibitors of the enzyme. Ideally, this type of chaperone should bind alpha‐galactosidaseA in the endoplasmic reticulum, stabilize it during its transport to lysosomes and dissociate in the acidic compartment where the enzyme encounters its substrate and exerts its biological activity.

**Figure 1:**
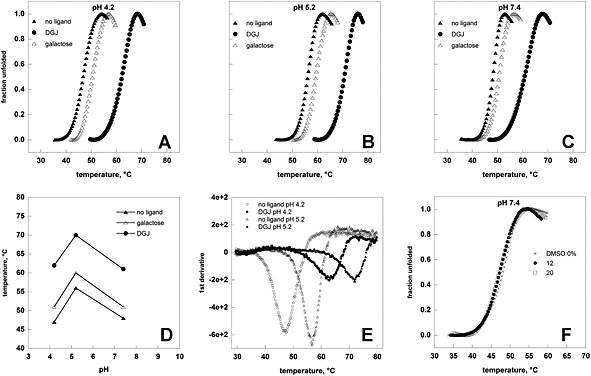
Heat‐induced melting profiles of lysosomal alpha‐galactosidaseA (Fabrazyme®) recorded by thermal shift. Temperature melting profiles of Fabrazyme® were recorded at different pH values ( 4.2 in panel A, 5.2 in panel B, 7.4 in panels C and F) and the experiment was conducted with no ligand, in the presence of 100 mM galactose or 1‐deoxy‐galactonojirimycin (DGJ) 40 μM (panels A, B, C) or DMSO up to 20% (panel F). The protein (0.5 mg/mL) was heated from 25 to 85° at 0.5°C/min in the presence of Sypro Orange. Data were shown as normalized curves (A, B, C, F) or first derivatives of raw data (E). Panel D shows T_0.5_ derived from the curves shown in panels A, B, or C as a function of pH.

**Figure 2:**
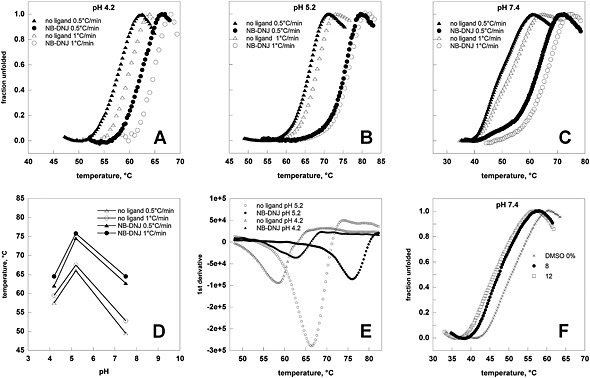
Heat‐induced melting profiles of lysosomal alpha‐glucosidase (Myozyme®) recorded by thermal shift. Temperature melting profiles of Myozyme® were recorded at different pH values (4.2 in panel A, 5.2 in panel B, 7.4 in panels C and F) and the experiment was conducted with no ligand, in the presence of N‐butyldeoxygalactonojirimycin (NB‐DNJ) 40 μM (panels A, B, C) or DMSO up to 12% (panel F). The protein (0.5 mg/mL) was heated from 25 to 85° at 0.5°C/min or 1°C/min in the presence of Sypro Orange. Data were shown as normalized curves (panels A, B, C, F) or first derivatives of raw data (E). Panel D shows T_0.5_ derived from the curves shown in panels A, B or C as a function of pH.

In Figure 2, panels A, B, or C, we show the melting profiles of Myozyme® recorded at 0.5°C/min or 1.0°C/min in the presence or in the absence of a ligand (0.04 mM NB‐DNJ (SIGMA, Milan, Italy)). Contrary to the case represented by Fabrazyme®, results depend on the scanning velocity and the apparent higher stability is inferred from the curves recorded at 1.0°C/min. Nonetheless results are reproducible provided that data are collected at the same scanning velocity (1.0°C/min), as we observed comparing T_0.5_ measure by Flanagan *et al*. (T_0.5_=53°C neutral pH no ligand; T_0.5_=68°C acidic lysosomal pH no ligand)^17^ and by us (T_0.5_=53°C neutral pH no ligand; T_0.5_=68°C acidic lysosomal pH no ligand). In relative terms results are robust since the ΔT_0.5_ observed with or without the ligand at the three pHs is the same and do not depend on the scanning velocity. The experiments carried out at various pHs consents to demonstrate that NB‐DNJ stabilizes Myozyme® better at neutral pH than at acidic pHs (Figure 2, panel D). A possible explanation could be that a hydrogen‐bond acceptor, which is protonated at low pH, plays a role in the binding at neutral pH. As a consequence of this, NB‐DNJ might preferentially stabilize alpha‐glucosidase mutants in the endoplasmic reticulum than in the lysosomes, leaving the enzyme more available to act on the substrate.

So far we have shown normalized melting profiles (Figure 1, panels A,B,C and Figure 2, panels A,B,C) obtained with the previously described equation fu(T) = f(T)‐fn/fd‐fn. But in order to evaluate the stabilizing effect of ligands it is sufficient to process raw data with software that are provided with any Real Time PCR equipment. In Figure 1, panel E and Figure 2, panel E we show the curves that can be obtained calculating the first derivatives of raw data; the temperature at which the minimum is observed corresponds with T_0.5_ and differences between the two values are negligible if compared with variability, on average ±1°C, among our replicas.

High throughput screenings are often carried out in the presence of dimethyl sulfoxide (DMSO). The effect of the solvent on Fabrazyme® (Figure 1, panel F) and Myozyme® (Figure 2, panel F) is different for the two enzymes suggesting that it must be evaluated case by case when planning a screening.

In conclusion, TSA offers advantages over other methods, differential scanning calorimetry,^15^ isothermal titration calorimetry,^18^ circular dichroism,^16^ chemical induced denaturation followed by intrinsic fluorescence detection^16^ or limited proteolysis,^19^ because it can process many samples containing a small amount of protein at the same time, is fast and requires equipment which is largely available in the majority of biomedical laboratories and processing of data is very simple. The method is robust, reproducible and versatile. It should be considered that the effect of scanning rates and the effect of solvents can vary case by case. In relative terms, binding of ligands and stabilization of the protein target can be detected with fast scanning rates, which are commonly employed and might be preferable for high throughput screening plans when thousand of molecules must be tested.
